# Interleukin 6 as a marker of severe bacterial infection in children with sickle cell disease and fever: a case–control study

**DOI:** 10.1186/s12879-021-06470-4

**Published:** 2021-08-03

**Authors:** Elena María Rincón-López, María Luisa Navarro Gómez, Teresa Hernández-Sampelayo Matos, David Aguilera-Alonso, Eva Dueñas Moreno, Jesús Saavedra-Lozano, Begoña Santiago García, María del Mar Santos Sebastián, Marina García Morín, Cristina Beléndez Bieler, Jorge Lorente Romero, Elena Cela de Julián, F-DREP Study Group, Alicia Hernanz Lobo, Carmen Garrido Colino, Jorge Huerta Aragonés, Cristina Mata Fernández, Eduardo Bardón Cancho, Concepción Míguez Navarro, Andrea Mora Capín, Rafael Marañón Pardillo, Arístides Rivas García, Paula Vázquez López, José Luis Jiménez Fuentes, Maria Ángeles Muñoz Fernández, Rosario Zamarro Arranz

**Affiliations:** 1 Department of Pediatrics, Pediatric Infectious Diseases Unit, Hospital General Universitario Gregorio Marañón, Instituto de Investigación Sanitaria Gregorio Marañón, c/O’Donnell 48-50, 28009 Madrid, Spain; 2 PhD Program in Medicine, Universidad Complutense de Madrid, Madrid, Spain; 3 Universidad Complutense de Madrid, Madrid, Spain; 4 Department of Pediatrics, Pediatric Hematology and Oncology Unit, Hospital General Universitario Gregorio Marañón, Madrid, Spain; 5 Department of Pediatrics, Pediatric Emergency Unit, Hospital General Universitario Gregorio Marañón, Madrid, Spain

**Keywords:** Sickle cell disease, Infection, Fever, Cytokines, Interleukin-6

## Abstract

**Background:**

Etiological diagnosis of fever in children with sickle cell disease (SCD) is often challenging. The aim of this study was to analyze the pattern of inflammatory biomarkers in SCD febrile children and controls, in order to determine predictors of severe bacterial infection (SBI).

**Methods:**

A prospective, case–control study was carried out during 3 years, including patients younger than 18 years with SCD and fever (cases) and asymptomatic steady-state SCD children (controls). Clinical characteristics and laboratory parameters, including 10 serum proinflammatory cytokines (IL-1β, IL-2, IL-4, IL-6, IL-8, IL-10, IL-12p70, IL-17a, IFN-γ and TNF-α) and comparisons among study subgroups were analyzed.

**Results:**

A total of 137 patients (79 cases and 58 controls) were included in the study; 78.5% males, median age 4.1 (1.7–7.5) years. Four cases were diagnosed with SBI, 41 viral infection (VI), 33 no proven infection (NPI) and 1 bacterial-viral coinfection (the latter excluded from the subanalyses). IL-6 was significantly higher in patients with SBI than in patients with VI or NPI (163 vs 0.7 vs 0.7 pg/ml, p < 0.001), and undetectable in all controls. The rest of the cytokines analyzed did not show any significant difference. The optimal cut-off value of IL-6 for the diagnosis of SBI was 125 pg/mL, with high PPV and NPV (PPV of 100% for a prevalence rate of 5, 10 and 15% and NPV of 98.7%, 97.3% and 95.8% for those prevalences rates, respectively).

**Conclusion:**

We found that IL-6 (with a cut-off value of 125 pg/ml) was an optimal marker for SBI in this cohort of febrile SCD children, with high PPV and NPV. Therefore, given its rapid elevation, IL-6 may be useful to early discriminate SCD children at risk of SBI, in order to guide their management.

**Supplementary Information:**

The online version contains supplementary material available at 10.1186/s12879-021-06470-4.

## Background

Febrile episodes are common in children with sickle cell disease (SCD), often with challenging etiological diagnosis. These patients are at risk of severe bacterial infections (SBI), although they have become less frequent in high-income countries in recent years [1–5]. Therefore, it would be important to find biomarkers that could help distinguish the etiology of these febrile episodes early in order to guide antimicrobial therapy. Several biomarkers [white blood count (WBC), neutrophils, C-reactive protein (CRP) and procalcitonin, among others] have been studied in SCD patients with bacteremia and other bacterial infections with variable results [1, 6–9]. Serum cytokines have also been studied in steady-state SCD patients and those with vaso-occlusive crisis (VOC) [10–15], but they have not been well studied in SCD patients with an infection.

The aim of this study was to analyze the pattern of inflammatory biomarkers, including pro-inflammatory cytokines, in SCD children with fever of different etiologies, in order to find useful parameters to discriminate the patients at risk of SBI. Since SBI has become less frequent in well-controled SCD children in recent years, this could help to guide the management of these patients, reducing unnecessary antibiotic treatments and hospital admissions.

## Material and methods

### Study design

We conducted a prospective, single-center, case–control study with patients younger than 18 years with SCD from June 2015 to June 2018. Children with SCD and fever were eligible to participate in the study as cases and asymptomatic steady-state SCD children were included as controls. Exclusion criteria of cases and controls included age older than 18 years, history of hematopoietic stem cell transplantation, incomplete diagnostic tests and patients whose parents or legal guardians did not sign the informed consent form for the study. Controls were also excluded if they had had a febrile episode in the previous month. The study received approval from the Institutional Review Board.

### Study setting

This study was conducted at the Hospital General Universitario Gregorio Marañón (HGUGM) in Madrid, Spain, a tertiary hospital with 120 pediatric beds and around 7000 pediatric inpatient admissions per year. HGUGM is a national reference center for children with SCD in Spain, with about 300 SCD pediatric patients upon follow-up.

### Study protocol and definitions

Patients included in the study as cases were those with an axillary temperature of 38ºC or more, documented at home or within the first 24 h of hospitalization. Controls were asymptomatic steady-state SCD patients recruited during their follow-up attendance in the Pediatric Hematology clinic. Patients recruited as cases could also be included in the study as controls if at least one month had passed since their last fever episode. The following samples were collected in all cases upon arrival of patients to the emergency department (or at the time of onset of fever if it appeared during the first 24 h of hospitalization): blood tests (including complete blood count, biochemistry, CRP and procalcitonin), blood cultures and nasopharyngeal samples for viral detection by a multiplex polymerase chain reaction **(**multiplex-PCR) assay [including influenza A, B and C, respiratory syncytial virus (RSV), human metapneumovirus, adenovirus, human bocavirus, rhinovirus, enterovirus, human parainfluenza virus and coronavirus]. Other diagnostic tests and the patients’ management were performed under the criteria of the treating physicians. In all cases and controls a blood sample was collected and stored at the HGUGM Biobank for subsequent cytokine analysis. This analysis was performed by the DIAplex Human Th1 / Th2 / Inflammation kit (bioNova científica S. L., Spain), a multiplexed fluorescent bead-based immunoassay for the quantification of multiple human cytokines in serum and culture supernatants by flow cytometry, including the following: interleukin (IL)-1β, IL-2, IL-4, IL-6, IL-8, IL-10, IL-12p70, IL-17a, interferon-γ (IFN-γ) and tumor necrosis factor- α (TNF-α). The limit of detection for the different cytokines was: IL-1β 3.5 pg/mL, IL-2 12.4 pg/mL, IL-4 4.3 pg/mL, IL-6 1.4 pg/mL, IL-8 1.3 pg/mL, IL-10 1.7 pg/mL, IL-12p70 3.4 pg/mL, IL-17a 8.7 pg/mL, TNFα 9.8 pg/mL and IFN-γ 0.8 pg/mL. For statistical analysis, when a single value was non-detectable, it was considered to be half the limit of detection of that cytokine.

SBI was defined as bacteremia, meningitis, pneumonia, osteomyelitis, urinary tract infection (UTI) or any other severe infection with the identification of a plausible microorganism in a normally sterile site (only confirmed SBI were included in this group). For the diagnosis of UTI, pyuria in urine analysis was required in addition to the positive urine culture with a threshold level of ≥ 10^5^ CFU/ml. Viral infection (VI) was defined as a positive result in the multiplex-PCR from respiratory samples. Patients with a bacterial infection and a viral detection in the respiratory sample at the same time were classified in the group of bacterial-viral coinfections and excluded from the subanalyses. Patients with no proven infection (NPI) were those not included in the previous groups.

### Variables evaluated

Data were collected in a database specifically designed for the study. Data related to cases and controls included baseline characteristics such as age, gender, SCD type, diagnostic method, parents’ continent of origin, immunization status (including vaccination against *Haemophilus influenzae* type b*, Streptococcus pneumoniae* and *Neisseria meningitidis* serogroups A, B, C, W, Y), penicillin prophylaxis, treatment with hydroxyurea, other treatments, long-term central venous catheter (CVC), splenectomy and history of previous hospital admissions. Clinical data during the episode (only in cases) included duration of fever before admission, maximum axillary temperature during the episode, respiratory symptoms, hemodynamic instability (defined as hypotension or persistent tachycardia requiring fluid resuscitation or inotropic drugs) and hypoxemia (< 92%). Laboratory parameters included hemoglobin, WBC, neutrophils and platelets on admission, initial and maximum CRP and procalcitonin during the episode, blood cultures, as well as other cultures and viral tests. Other recorded data were inpatient/outpatient management, antimicrobial treatment, need for antibiotic change because of unfavorable clinical course, diagnosis of VOC or acute chest syndrome (ACS), duration of fever and of hospitalization, pediatric intensive care unit (PICU) admission and final outcome (hospital discharge or mortality). Cytokine levels in all cases and controls were also evaluated.

### Statistical analysis

Qualitative variables were expressed as frequencies or rates. Quantitative variables were reported as means (standard deviation) or medians [interquartile range (IQR)], according to their distribution*.* The χ^2^ test was used to compare categorical variables; Yates correction was applied to any contingency table and Fisher’s exact test was used when at least one cell had a value < 5. T-test and ANOVA were used to compare the means of normally distributed variables whereas Mann–Whitney U test and Kruskal–Wallis test were performed to compare the medians of variables not normally distributed. Receiver operating characteristics (ROC) curves and area under the ROC curve (AUC) were calculated for significant parameters. The optimal cut-off point of each biomarker was selected using the Youden Index, based on the maximum sum of sensitivity and specificity. Sensitivity, specificity, positive predictive values (VPP) and negative predictive values (NPV) were calculated according to various possible prevalence rates (PR) of SBI. Two-tailed p values < 0.05 were considered statistically significant. Statistical analysis was performed using IBM SPSS Statistics software version 22.0 (IBM Corp., Armonk, N.Y., USA).

## Results

**Fig. 1:**
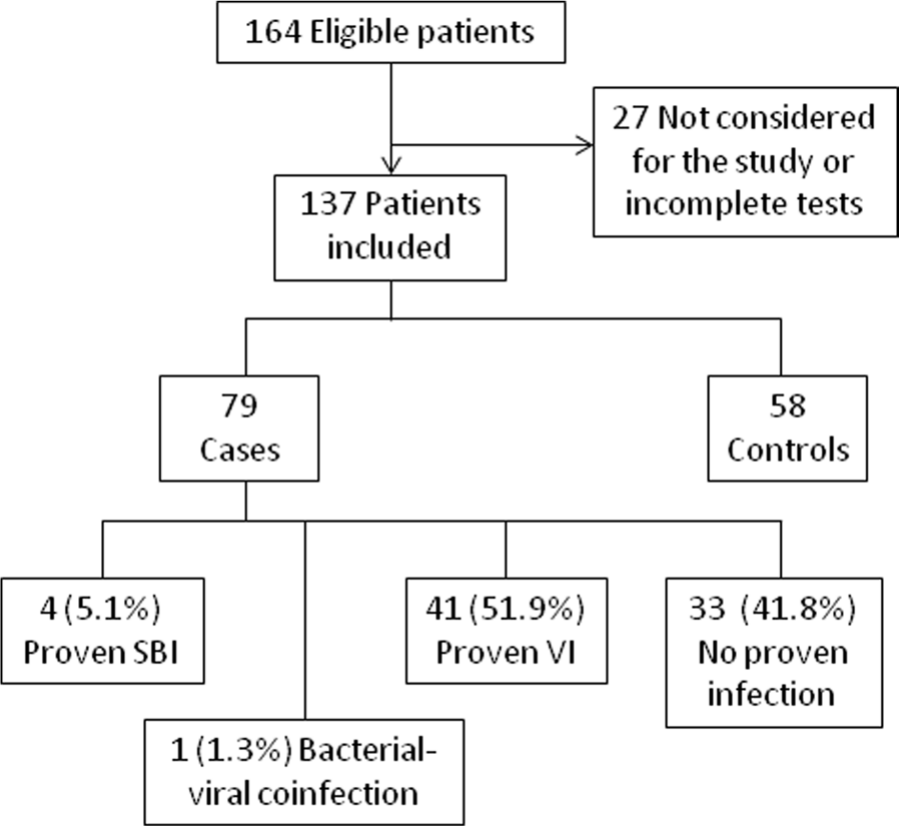
Flow diagram

A total of 137 patients were included in the study: 79 cases and 58 controls (Fig. 1). Median age of the patients was 4.1 (1.7–7.5) years for cases and 5.2 (2.1–9.3) for controls (p = 0.193), with a higher percentage of males in the group of cases (78.5% vs 62.1%, p = 0.035). The majority of the children were born in Spain and had been diagnosed by newborn screening, while their parents were predominantly from Africa and South/Central America. Most patients were appropriately immunized and receiving penicillin prophylaxis, while approximately half of them were on hydroxyurea treatment. Baseline characteristics of cases and controls are summarized in Table 1.

**Table 1:** Baseline characteristics of patients

	Cases (n = 79)	Controls (n = 58)	p value
Age in years [m (IQR)]	4.1 (1.7–7.5)	5.2 (2.1–9.3)	0.193
Male [no. (%)]	62 (78.5)	36 (62.1)	**0.035**
Genotype [no. (%)]			
SS	68 (86.1)	52 (89.7)	0.592
SC	5 (6.3)	4 (6.9)	
Sβ*-thalassemia*	6 (7.6)	2 (3.4)	
Newborn screening [no. (%)]	72 (91.1)	47 (81)	0.084
Parents’ origin [no. (%)]			
Africa	33 (41.8)	42 (72.4)	**0.001**
America	45 (57)	14 (24.1)	
Other	1 (1.3)	2 (3.4)	
Complete vaccination status [no. (%)]	70 (88.6)	48 (85.7)	0.618
Penicillin prophylaxis [no. (%)]	76 (98.7)	53 (91.4)	0.084
Hydroxyurea [no. (%)]	39 (49.4)	33 (56.9)	0.132
Vitamin D supplementation [no. (%)]	77 (97.5)	56 (96.6)	0.753
Splenectomy [no. (%)]	8 (10.1)	4 (6.9)	0.509
Central venous catheter [no. (%)]	18 (22.8)	4 (6.9)	**0.012**
Hypertransfusional regimen [no. (%)]	9 (11.4)	2 (3.4)	0.117
No. of previous hospital admissions [m (IQR)]	6 (2–10.5)	5 (2–9)	0.138

m (IQR) = median (interquartile range). No. = number

Variables with significant differences (p value < 0.05) are highlighted in bold font

Four out of 79 cases (5.1%) were diagnosed with SBI: two of them were catheter-related bacteremia (caused by *Staphylococcus aureus* and *Enterobacter cloacae*), one *Streptococcus pneumoniae* (serotype 9 N) bacteremic pneumonia and one *Escherichia coli* UTI. Another patient was also diagnosed with *Escherichia coli* UTI, but she had a viral detection in a respiratory sample at the same time (influenza B) and, therefore, she was considered a bacterial-viral coinfection and excluded from the subanalyses. A virus was detected by multiplex-PCR in 41/79 (51.9%) of the respiratory samples. The most frequently detected virus was influenza (A or B) in 23.3% of the cases, followed by rhinovirus in 20.9%, adenovirus in 14% and enterovirus in 11.6%. When comparing the baseline characteristics among cases with SBI, VI and the rest of patients with NPI (Additional file 1: Table S1), no statistically significant differences were found, except for the presence of CVC and hypertransfusional regimen, which were more frequent in the SBI group (50% vs 9.8% vs 36.4%, p = 0.011 and 50% vs 4.9% vs 15.2%, p = 0.018, respectively).

Data on clinical presentation, laboratory parameters and outcome during the febrile episodes in all cases, and comparisons among groups, are summarized in Table 2. Most patients were hospitalized (81%) and received at least one dose of empiric antibiotic treatment (96.2%). Three patients (3.8%) required PICU admission and no patient died. Patients with SBI had more often hemodynamic instability compared to patients with VI or NPI (25% vs 2.4% vs 0, respectively; p = 0.012) and significantly higher inflammatory parameters (neutrophils, CRP and procalcitonin). They required more frequently a change in the antibiotic therapy (75% vs 7.5% vs 15.6%; p = 0.001) and longer hospital admissions (7.5 vs 3 vs 5 days; p = 0.013). Upper respiratory symptoms were more frequent in patients with VI compared to patients with SBI or NPI (80.5% vs 50% vs 42.4%, respectively; p = 0.003).

**Table 2:** Characteristics of cases during the febrile episode and differences among study subgroups

	All cases (n = 79)	SBI (n = 4)	VI (n = 41)	NPI (n = 33)	p value
*Clinical presentation*					
Previous days of fever [m (IQR)]	1 (1–1)	1 (1–2)	1 (1–2)	1 (1–1)	0.459
Max. temperature [m (IQR)]	38.8 (38.4–39.1)	39.1 (38.9–39.2)	38.7 (38.3–39.2)	38.8 (38.5–39)	0.430
Upper respiratory symptoms [no. (%)]	49 (62)	2 (50)	33 (80.5)	14 (42.4)	**0.003**
Hemodynamic instability [no. (%)]	2 (2.5)	1 (25)	1 (2.4)	0	**0.012**
Hypoxemia [no. (%)]	10 (12.7)	0	4 (9.8)	6 (18.2)	0.410
*Laboratory parameters*					
Initial hemoglobin g/dl [m (IQR)]	8.5 (7.5–9.5)	9 (8.4–9.5)	8.5 (7.4–9.7)	8.5 (7.5–9.5)	0.681
Initial WBC × 10^9^ /L [m (IQR)]	13.8 (9.7–21.1)	20.7 (17.6–31.4)	11.8 (8.9–21.2)	13.9 (10.9–19.8)	0.160
Initial neutrophils × 10^9^ /L [m (IQR)]	8.1 (5.1–13.8)	16.8 (13.9–26.7)	7 (3.7–13.3)	7.8 (6.1–12.5)	**0.039**
Initial CRP mg/dl [m (IQR)]	2 (0.4–5.9)	9.6 (7.8–15.1)	1.1 (0.4–3.4)	3.1 (0.6–6)	**0.007**
Max. CRP mg/dl (n = 56) [m (IQR)]	4 (1.1–10.1)	13.5 (12.3–17.9)	3.3 (1–7.2)	5.2 (1.2–9.4)	**0.047**
Initial PCT ng/ml [m (IQR)]	0.3 (0.2–0.6)	2 (0.8–2.9)	0.4 (0.2–0.6)	0.2 (0.1–0.4)	**0.036**
Max. PCT ng/ml (n = 40) [m (IQR)]	0.5 (0.2–1.4)	3.1 (1.8–18.9)	0.6 (0.3–1.3)	0.3 (0.2–0.5)	**0.031**
*Outcome*					
Hospital admission [no. (%)]	64 (81)	4 (100)	29 (70.7)	30 (90.9)	0.055
Antibiotic treatment [no. (%)]	76 (96.2)	4 (100)	39 (95.1)	32 (97)	0.845
Need for antibiotic change [no. (%)]	12 (15.2)	3 (75)	3 (7.5)	5 (15.6)	**0.001**
Final diagnosis of ACS [no. (%)]	13 (16.5)	1 (25)	5 (12.2)	7 (21.2)	0.527
Final diagnosis of VOC [no. (%)]	7 (8.9)	0	1 (2.4)	6 (18.2)	0.051
PICU admission [no. (%)]	3 (3.8)	0	1 (2.4)	2 (6.1)	0.665
Total days of fever [m (IQR)]	2 (1–4)	2.5 (2–4)	2 (2–3)	2 (1–4)	0.749
Days of admission [m (IQR)]	4 (2–6)	7.5 (5.5–8.5)	3 (0.5–5)	5(2.5–6)	**0.013**

*m (IQR)*  median (interquartile range), *no.*  number, *Max.*  maximum value during the episode, *WBC*  white blood count, *CRP*  C-reactive protein, *PCT*  procalcitonin, *ACS*  acute chest syndrome, *VOC*  vaso-occlusive crisis

Variables with significant differences (p value < 0.05) are highlighted in bold font

### Analysis of proinflammatory cytokine levels and other biomarkers

**Fig. 2:**
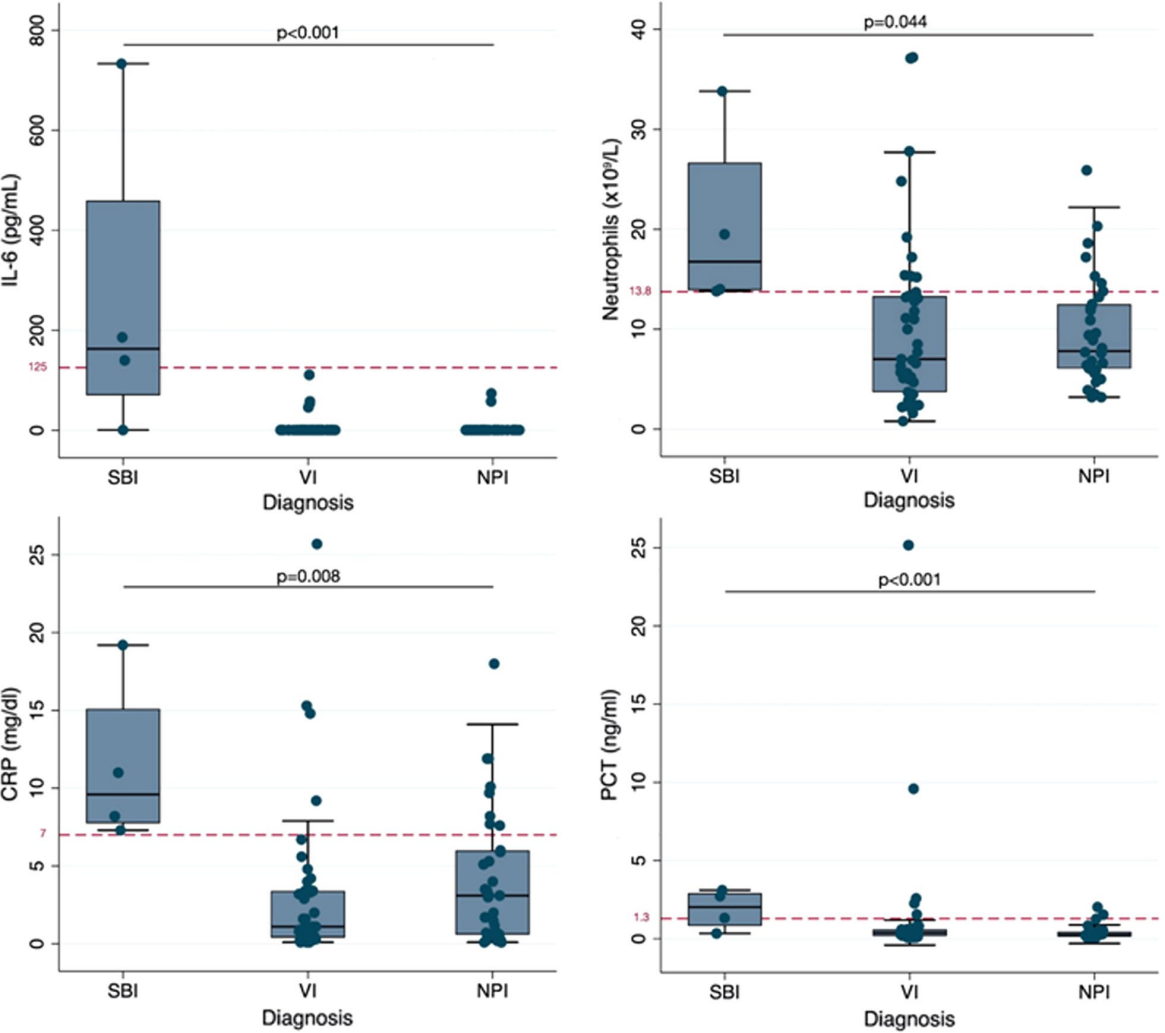
Biomarkers’ values in the study subgroups and optimal cut-off points

IL-6 was significantly higher in patients with SBI than in those with VI or NPI [median (IQR) pg/ml: 163 (70.4–459.5) vs 0.7 (0.7–0.7) vs 0.7 (0.7–0.7), respectively; p < 0.001], being undetectable in all controls. To note, the patient with the highest level of IL-6 was the case of confirmed bacterial pneumonia (733 pg/ml). However, in the patient with the bacterial-viral coinfection, IL-6 was undetectable. Figure 2 represents the values of IL-6 and other significant biomarkers (neutrophils, CRP and PCT) of the study subgroups, including the optimal cut-off point for each parameter calculated by the Youden index. IL-12p70 was not detected in any of the cases or controls. The rest of the cytokines analyzed (IL-1β, IL-2, IL-4, IL-8, IL-10, IL-17a, TNFα, IFN-γ) did not show any significant difference between cases and controls or among different groups of cases (Table 3). No differences were found in any of the cytokines analyzed between the cases diagnosed with VOC and the rest of the patients. None of the cytokine levels significantly correlated with possible confounders as the age or the number of previous admissions.

**Table 3:** Cytokines values and comparisons among study subgroups

	Cases (n = 79)	Controls (n = 58)	p value	SBI (n = 4)	VI (n = 41)	NPI (n = 33)	p value
IL-1β pg/ml	33 (1.8–242.5)	29.5 (1.8–212)	0.861	57.9 (1.8–190)	37 (1.8–289)	33 (1.8–166)	0.828
IL-2 pg/ml	6.2 (6.2–6.2)	6.2 (6.2–6.2)	0.565	ND	6.2 (6.2–6.2)	6.2 (6.2–6.2)	0.763
IL-4 pg/ml	2.2 (2.2–85.5)	2.2 (2.2–91)	0.509	14.6 (2.2–149.5)	2.2 (2.2–69)	2.2 (2.2–226)	0.805
IL-6 pg/ml	0.7 (0.7–0.7)	ND	**0.008**	163 (70.4–459.5)	0.7 (0.7–0.7)	0.7 (0.7–0.7)	** < 0.001**
IL-8 pg/ml	41 (30–74)	36 (30–52)	0.191	49 (32.5–68.5)	44 (30–99)	38 (30–72)	0.919
IL-10 pg/ml	0.9 (0.9–73)	0.9 (0.9–34)	0.218	17.4 (0.9–116.5)	9.9 (0.9–94)	0.9 (0.9–58)	0.561
IL-12p70 pg/ml	ND	ND	–	ND	ND	ND	–
IL-17a pg/ml	4.4 (4.4–266)	44 (4.4–233)	0.828	53.7 (4.4–263.5)	4.4 (4.4–248)	35 (4.4–284)	0.912
TNF-α pg/ml	59.0 (11–112)	50.5 (23–92)	0.674	24.5 (14–52.5)	64 (26–156)	64 (4.9–106)	0.357
IFN-γ pg/ml	41 (0.4–152)	34 (0.4–97)	0.324	0.4 (0.4–20.7)	57 (0.4–203)	41 (0.4–132)	0.201

Variables with significant differences (p value < 0.05) are highlighted in bold font

ND  non-detectable in any of the patients of the subgroup

The AUC of IL-6 to discriminate confirmed SBI was 0.87 (95% CI 0.77–0.93), similarly to the other significant parameters [neutrophils 0.87 (0.78–0.94); CRP 0.89 (0.81–0.96); procalcitonin 0.84 (0.73–0.91)]. The optimal cut-off value of IL-6 for the diagnosis of SBI was 125 pg/mL. Data of sensibility, specificity, PPV and NPV (the last two according to various possible PR of SBI) of IL-6 and other significant biomarkers are represented in Table 4. IL-6 had very high values of PPV and NPV, NPV values being similar to other biomarkers, with significantly higher PPV values (PPV of 100% for a PR of 5, 10 and 15%; NPV of 98.7%, 97.3% and 95.8% for a PR of 5, 10 and 15%, respectively).

**Table 4:** Diagnostic tests of IL-6 and other significant biomarkers for the prediction of SBI

Biomarker	Cut-off	Sensitivity % (95% CI)	Specificity % (95% CI)	PPV % (95% CI)	NPV % (95% CI)
IL-6	125 pg/ml	75 (21.9–98.7)	100 (93.9–99.9)	PR 5%: 100 (56.6–100) PR 10%: 100 (56.6–100) PR 15%: 100 (56.6–100)	PR 5%: 98.7 (93.3–99.8) PR 10%: 97.3 (86.8–99.5) PR 15%: 95.8 (80.6–99.2)
Neutrophils	13.8 × 10^9^/L	100 (39.8–100)	77.3 (66.2–86.2)	PR 5%: 18.8 (13.3–26.1) PR 10%: 32.9 (24.4–42.7) PR 15%: 43.8 (33.9–54.2)	PR 5%: 100 (87.8–100) PR 10%: 100 (87.8–100) PR 15%: 100 (87.8–100)
CRP	7 mg/dl	100 (39.8–100)	82.7 (72.2–90.4)	PR 5%: 23.3 (15.6–33.2) PR 10%: 39.1 (28.1–51.2) PR 15%: 50.4 (38.3–62.5)	PR 5%: 100 (92–7-100) PR 10%: 100 (92–7-100) PR 15%: 100 (92–7-100)
Procalcitonin	1.3 ng/ml	75 (19.4–99.4)	82.7 (72.2–90.4)	PR 5%: 18.5 (9–7-32.6) PR 10%: 32.5 (18.5–50.5) PR 15%: 43.3 (26.5–61.8)	PR 5%: 98.4 (92–99.7) PR 10%: 96.7 (84.5–99.4) PR 15%: 94.9 (77.4–99)

## Discussion

In this prospective, case–control study we compared the clinical characteristics and laboratory parameters, including 10 serum proinflammatory cytokines, in SCD children with fever of different etiologies and asymptomatic steady-state controls. We found that IL-6 was a very good biomarker for the diagnosis of SBI in these patients, with similar NPV to other biomarkers and better PPV. To our knowledge, this is the first study describing IL-6 as a marker of SBI in febrile SCD children.

Most of the children included in this study had been diagnosed with SCD by newborn screening, had a complete vaccination status and were receiving penicillin prophylaxis. In the group of SCD patients with fever, we found a low proportion of confirmed SBI (5.1%), similarly to other studies performed in high-income countries [1, 3–5]. However, in more than a half of the cases (52.6%) a virus was detected by multiplex-PCR from respiratory samples, as it has been observed in recent pediatric studies [16, 17]. In the remaining 42.8% of cases an infectious etiology was not confirmed. The only statistically significant differences among the baseline characteristics of study subgroups were a higher percentage of patients with CVC and on hypertransfusional regimen in the group of SBI, suggesting a poorer clinical baseline condition of these patients and also highlighting the presence of long-term CVC as a predisposing factor for bacteremia [6].

Despite the low proportion of confirmed SBI in this cohort, almost all patients received at least one dose of antibiotic (96.2%), in agreement to current guidelines [18–20].This high use of antibiotics emphasizes the importance of defining laboratory parameters that could early discriminate the etiology of fever in these patients, in order to optimize the antimicrobial prescription. Children with SBI presented more frequently with hemodynamic instability and had higher inflammatory parameters (neutrophils, CRP and procalcitonin) than the rest of the patients, similarly to what was described in other studies [1, 5–9]. We also found that children with SBI had significantly higher IL-6 levels when compared with those with VI, NPI and controls, with the highest value observed in the only patient with confirmed pneumococcal pneumonia. The optimal cut-off value of IL-6 for the diagnosis of SBI was 125 pg/ml, with a very high PPV (significantly better than other biomarkers) and NPV applying to different PR. In a setting with a PR of SBI of 5% in children with SCD (average PR in high-income countries [1, 4, 5]), the PPV would be 100% and NPV 98.7%. Therefore, we consider that IL-6 could be a very useful marker for the diagnosis of SCD patients with confirmed SBI.

IL-6 is an important factor in systemic and local immune response. It can be secreted by almost all stromal cells and cells of the immune system at an early stage of inflammation (with rapid elevation in 1–3 h), and further promote the synthesis and release of CRP by the liver [21, 22]. IL-6 has been previously described as a marker of different types of SBI (alone or in combination with other biomarkers) [23, 24] such as early-onset neonatal sepsis [25, 26], sepsis and septic shock [21, 23, 27] bacteremia in patients with febrile neutropenia [28, 29] or pneumococcal pneumonia [30–33]. The early elevation of IL-6 that occurs in the presence of SBI is an important advantage of this cytokine over other biomarkers.

In SCD patients, it has previously been described that IL-6 and other proinflammatory cytokines are elevated in asymptomatic steady-state patients when compared to healthy individuals [10–13]. However, in our study all controls (steady-state SCD patients) had undetectable levels of IL-6. A possible explanation for this could be that almost all children included in this study were receiving vitamin D supplements. According to the study performed by Adegoke et al., children with SCD had a maintained vitamin D deficiency with a high level of proinflammatory cytokines (IL-6, IL-8 and IL-18), which normalizes after vitamin D supplementation [34]. Other previous studies also found higher levels of IL-6 in patients with VOC [14, 15], a finding that we did not observe in our study.

The strengths of this study were its prospective design, the homogeneity of the patients and that it was carried out in a reference center for patients with SCD in Spain; therefore, this cohort may be quite representative of the pediatric population with SCD in high-income countries. However, it has also several limitations. Most importantly, the sample size was relatively small, with few cases of confirmed bacterial infection, and the categorization in several study groups might have decreased the statistical power. This fact could explain that we did not find statistically significant differences in the rest of the cytokines analyzed. In addition, due to the sample size, we did not perform a multivariate analysis, as it could potentially have an insufficient statistical power. Secondly, blood samples were collected regardless the previous duration of fever and serial samples were not collected, so it was not possible to evaluate the trend over time of the different biomarkers during the febrile episode. Finally, only confirmed SBI were included in the study to avoid bias in the analysis, but maybe some not-confirmed bacterial infections (e. g. pneumonia) were included in the NPI group.

## Conclusion

In this prospective study, we found that IL-6 (with an optimal cut-off of 125 pg/ml) was a good marker of SBI with a high PPV and NPV. Therefore, given its rapid elevation, IL-6 may be useful (alone or in combination with other biomarkers) to early discriminate SCD children at risk of SBI, in order to guide their management. In settings with a low PR of SBI, its use could help to reduce unnecessary antibiotic treatments and hospital admissions. These findings should be confirmed in larger cohorts and multicenter studies.

## Supplementary Information

**Additional file 1: Table S1.** Baseline characteristics of cases and comparisons among study subgroups.

## Data Availability

The data that support the findings of this study are available from the corresponding author but restrictions apply to the availability of these data, which were used under license for the current study, and so are not publicly available. Data are however available from the authors upon reasonable request and with permission of the corresponding author.

## Abbreviations

SCD: Sickle cell disease
SBI: Severe bacterial infection
VOC: Vaso-occlusive crisis
HGUGM: Hospital General Universitario Gregorio Marañón
CRP: C-reactive protein
Multiplex-PCR: Multiplex polymerase chain reaction
RSV: Respiratory syncytial virus
IL: Interleukin
IFN: Interferon
TNF: Tumor necrosis factor
UTI: Urinary tract infection
VI: Viral infection
NPI: No proven infection
CVC: Central venous catheter
ACS: Acute chest syndrome
PICU: Pediatric intensive care unit
IQR: Interquartile range
ROC: Receiver operating characteristic
AUC: Area under the ROC curve
PPV: Positive predictive value
NPV: Negative predictive value
PR: Prevalence rate
CI: Confidence interval
