# Supplementary material for: Interleukin 6 as a marker of severe bacterial infection in children with sickle cell disease and fever: a case–control study
Source: BMC Infect Dis. 2021 Aug 3;21:741. doi: 10.1186/s12879-021-06470-4 (PMC8329904; doi:10.1186/s12879-021-06470-4)
Supplement: Supplementary file 1 — Additional file 1: Table S1. Baseline characteristics of cases and comparisons among study subgroups. [file 12879_2021_6470_MOESM1_ESM.docx]

**TABLE S1. Baseline characteristics of cases and comparisons among study subgroups**

|  | **Proven SBI**  **(n=4)** | **Proven VI**  **(n=41)** | **NPI**  **(n=33)** | **p** |
| --- | --- | --- | --- | --- |
| **Age in years [m (IQR)]** | 4 (1.4-6) | 4.1 (1.5-7) | 4.1 (1.8-7.9) | 0.736 |
| **Male [no. (%)]** | 3 (75) | 35 (85.4) | 24 (72.7) | 0.398 |
| **Newborn screening [no. (%)]** | 4 (100) | 38 (92.7) | 29 (87.9) | 0.627 |
| **Genotype [no. (%)]**  **SS**  **SC**  **Sβ*-*thalassemia** | 4 (100)  0  0 | 34 (82.9)  5 (12.2)  2 (4.9) | 29 (87.9)  0  4 (12.1) | 0.182 |
| **Parents’ origin [no. (%)]**  **Africa**  **America**  **Other** | 4 (100)  0  0 | 14 (34.1)  26 (63.4)  2.4 | 15 (45.5)  18 (54.5)  0 | 0.116 |
| **Complete vaccination status [no. (%)]** | 4 (100) | 37 (90.2) | 28 (84.8) | 0.585 |
| **Penicillin prophylaxis [no. (%)]** | 4 (100) | 39 (97.5) | 32 (100) | 0.634 |
| **Hydroxyurea [no. (%)]** | 1 (25) | 22 (53.7) | 16 (48.5) | 0.330 |
| **Vitamin D** **supplementation [no. (%)]** | 4 (100) | 39 (95.1) | 33 (100) | 0.396 |
| **Splenectomy [no. (%)]** | 0 | 4 (9.8) | 4 (12.1) | 0.743 |
| **Central venous catheter [no. (%)]** | 2 (50) | 4 (9.8) | 12 (36.4) | **0.011** |
| **Hypertransfusional regimen [no. (%)]** | 2 (50) | 2 (4.9) | 5 (15.2) | **0.020** |
| **No. of previous hospital admissions [m (IQR)]** | 3.5 (2-6.5) | 8 (3-11) | 6 (2-11) | 0.614 |

NPI = no proven infection. m (IQR) = median (interquartile range). No. = number.

Variables with significant differences (p value < 0.05) are highlighted in bold font.
